# Hormone secretion predicts poor prognosis in lung neuroendocrine neoplasms

**DOI:** 10.1530/ERC-26-0142

**Published:** 2026-06-18

**Authors:** Ieva Lase, Eir Löfgren, Agnes Hegedus, Malin Grönberg, Staffan Welin, Eva Tiensuu Janson

**Affiliations:** ^1^ Department of Medical Sciences, Endocrine Oncology Unit, Uppsala University, Uppsala, Sweden; ^2^ Department of Immunology, Genetics and Pathology, Uppsala University and Uppsala University Hospital, Uppsala, Sweden

**Keywords:** neuroendocrine, lungs, serotonin, multiple, prognosis

## Abstract

Ectopic hormone secretion from neuroendocrine neoplasms in the lung (lung-NENs) is rare and poorly explored. Emerging evidence indicates that hormone-secreting NENs carry a worse prognosis. We hypothesised that ectopic hormone production and secretion in lung-NENs is a negative prognostic marker. A retrospective cohort of 167 patients with lung-NENs (121 females, median age: 68 (min–max: 15–85) years), excluding small cell lung cancer, was analysed for clinical and biochemical data and outcome. Tumour tissue was evaluated for serotonin and calcitonin expression. Among 127 patients with hormone measurements, 52 had increased levels, with 5-hydroxyindoleacetic acid (5-HIAA) predominating (*n* = 38), followed by calcitonin (*n* = 20). There was no correlation between serotonin expression in tumour tissue and elevated levels of 5-HIAA, while high calcitonin levels were strongly correlated with calcitonin expression in tumour samples (OR: 21.86, *P* = 0.002). Furthermore, diffuse calcitonin staining was associated with a shorter overall survival (OS) (*P* = 0.036). Hormone-secreting tumours had a significantly shorter OS than non-secreting tumours (62 vs 124 months, *P* = 0.024), and multiple hormone secretion further worsened survival (33 vs 69 vs 124 months, *P* = 0.002). In multivariate analysis, multiple hormone secretion remained an independent negative prognostic factor (*P* = 0.042). In conclusion, hormone secretion in lung-NENs is associated with a shorter OS, particularly in patients with multiple hormone secretion. Diffuse calcitonin expression in tumour tissue is an additional negative prognostic marker. We recommend routine hormone testing at baseline and at disease progression in metastatic lung-NEN patients and suggest a more intensified therapeutic strategy in cases with detectable hormone secretion.

## Introduction

Although well-differentiated neuroendocrine neoplasms in the lung (lung-NENs) are still considered rare tumours, their incidence is increasing (1). It is well known that lung-NENs can produce various ectopic hormones, such as ACTH, serotonin and calcitonin; however, the prevalence and prognostic significance of ectopic hormone production remain poorly understood.

Lung-NENs are classified by cell morphology, mitotic count/2 mm^2^ (MC) and necrosis in the primary tumours as follows: typical carcinoid (TC) with MC < 2 and no necrosis; atypical carcinoid (AC) with MC 2–10 and/or necrosis; large cell neuroendocrine carcinoma (LCNEC) with MC > 10 and large cell cytomorphology; and small cell lung cancer (SCLC) with MC > 10 and small cell cytomorphology (2). Since the classification of lung-NENs is an important prognostic factor and guides therapeutic decisions also in metastatic disease, accurate histopathological subtyping is clinically relevant. However, in small biopsy specimens, assessment of MC and identification of necrosis can be challenging (3). The recent WHO classification (2) introduced the term ‘carcinoid tumour/metastatic carcinoid not otherwise specified (NOS)’ for biopsies where TC cannot be distinguished from AC (4) and recommends including proliferation index Ki-67 in pathology reports (2). Some authors suggest using a Ki-67 index cut-off value of 5% for the prediction of overall survival (OS) (5, 6). Recent guidelines acknowledge a subgroup of highly proliferative AC with well-differentiated morphology but higher proliferation markers (MC > 10 and/or Ki-67 > 20%) (3, 7), corresponding to G3 gastroenteropancreatic neuroendocrine tumours. Lung-NENs represent 20–25% of all lung tumours with predominance of SCLC (20%), followed by LCNEC (3%), TC (2%) and AC (0.2%) (4, 8, 9).

The hormone secretion from NENs can be classified as topic or ectopic, depending on whether the hormone is normally secreted from the organ of origin of the tumour or not. Ectopic hormone secretion with clinical symptoms is defined as an endocrine paraneoplastic syndrome (EPNS). The prevalence of EPNS has not been comprehensively investigated; however, the most frequently reported forms of ectopic hormone secretion in lung-NENs are serotonin and ACTH. Serotonin is secreted into the circulation following a multistep enzymatic process in tumour cells and then metabolised in the liver to 5-hydroxyindoleacetic acid (5-HIAA). The reported incidences of the carcinoid syndrome in lung-NENs vary between 2 and 13% (10, 11). In ACTH-secreting tumours, proopiomelanocortin (POMC) is converted to ACTH or ACTH precursors through abnormal expression of the POMC gene, leading to hypercortisolaemia (12, 13). ACTH secretion, causing ectopic Cushing’s syndrome (ECS), is present in 1–6% of lung-NENs (10). Meta-analyses of publications concerning calcitonin-secreting lung-NENs have shown that most reports focus on hypercalcitoninaemia in SCLC with only a limited number addressing calcitonin secretion in TC or AC (14, 15). Hypercalcitoninaemia in lung-NENs may be more common than previously recognised, likely due to the absence of specific clinical symptoms that would lead to measurement of calcitonin. In addition, synchronous secretion of other hormones, including serotonin, whose associated symptoms, flushing and diarrhoea, are usually recognised as related to serotonin secretion rather than calcitonin may also play a role (14, 16).

There is increasing evidence that hormone-producing NENs, particularly with multiple hormone secretion, carry a worse prognosis (17, 18, 19, 20). However, studies on EPNS impact on survival in lung-NENs are few and mostly focused on ECS (21, 22). To our knowledge, no studies have systematically investigated the prevalence and prognostic implication of serotonin and calcitonin production and secretion in lung-NENs, nor examined the relationship between immunohistochemical (IHC) expression of these hormones and circulating levels. Importantly, several studies described a potential stimulatory effect of serotonin on cancer cell proliferation, invasion, dissemination and tumour angiogenesis (23, 24, 25) and there is some evidence suggesting a role for calcitonin as a pro-angiogenic factor (26, 27).

The aim of this study was to analyse a cohort of lung-NENs to investigate the association between IHC marker expression and circulating levels of calcitonin and serotonin, and to evaluate their prognostic significance. We hypothesised that increased hormone production may be associated with worse survival outcome.

## Materials and methods

### Patients

A total of 167 patients with pulmonary NENs were identified from our databases of all patients referred to the Department of Endocrine Oncology, Uppsala University Hospital, between 1 January 2010 and 30 April 2025. Patients with SCLC were not included, since these tumours are not treated at our centre. In patients with elevated 5-HIAA levels, small intestinal NET was excluded by ^68^Ga-DOTATOC-PET/CT, and there was no radiological evidence of medullary thyroid carcinoma in patients with elevated calcitonin levels. Ectopic ACTH secretion was confirmed by the clinical and biochemical findings together with exclusion of a pituitary origin by pituitary MRI and inferior petrosal sinus sampling.

### Biochemical data

Multiples of upper normal limit (UNL) were used when describing 5-HIAA levels since both urine and serum assays were used with a UNL of 50 μmol/day and 123 nmol/L, respectively. Calcitonin levels were described as median with UNL 1.9 pmol/L for females and 2.8 pmol/L for males. Data on hormone levels were collected at diagnosis and during follow-up. Tumours were defined as functioning or non-functioning depending on the presence or absence of hormone-related symptoms and elevated circulating hormone levels. When comparing tumours with 5-HIAA and calcitonin secretion, tumours secreting ACTH in parallel were excluded. Unless otherwise specified, patients who were radically operated without recurrence and those without available hormone measurements were excluded from analysis of the impact of hormone secretion on overall survival (OS).

### Immunohistochemistry

The cohort was classified according to the latest WHO classification of thoracic tumours from 2021 (2) if the patient was operated on for the primary tumour (TC, AC and LCNEC). When biopsy material was used for diagnostic purposes, well-differentiated tumours were classified as AC in case of necrosis, MC > 2 or Ki-67 ≥ 10%, while the remaining well-differentiated tumours were classified as ‘carcinoid NOS’. Ki-67 was calculated in tumour material obtained at diagnosis.

**Figure 1:**
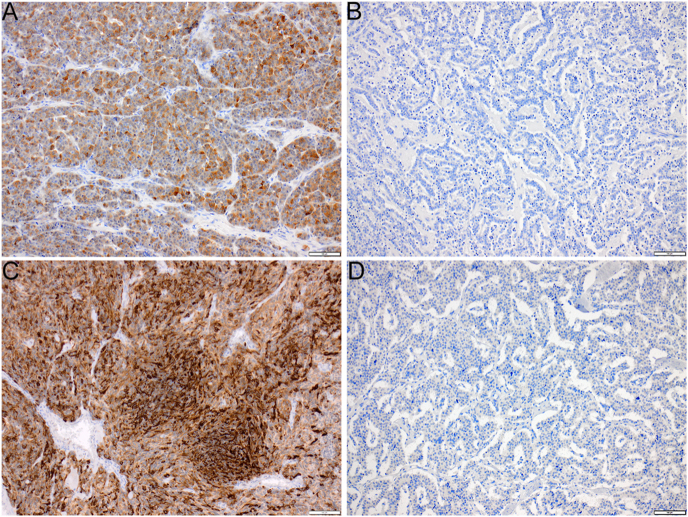
Representative pictures from immunohistochemical staining of serotonin and calcitonin in neuroendocrine neoplasms of the lung (typical carcinoids). Tumour positive for serotonin (A) and calcitonin (C). Tumour negative for serotonin (B) and calcitonin (D). Total magnification: 100×, scale bar: 100 μm. A full colour version of this figure is available at https://doi.org/10.1530/ERC-26-0142.

Formalin-fixed, paraffin-embedded (FFPE) sections obtained from retrospectively collected tumour specimens were immunohistochemically stained for serotonin and calcitonin using fully automated protocols on the Dako Omnis Autostainer (Agilent Technologies/Dako, Denmark), provided that sufficient tissue was available. The antibodies used included a mouse monoclonal antibody for serotonin (clone 5HT-H209; Dako, Denmark; Cat. No. M0758; dilution 1:300, antibody incubation for 20 min) and a rabbit polyclonal ready-to-use antibody for calcitonin (Dako, Denmark; Cat. No. GA515, dilution: ready to use, antibody incubation for 15 min). Positive controls were small intestinal NET for serotonin and medullary thyroid carcinoma for calcitonin. All slides underwent a two-step evaluation: an initial review by the first author and a subsequent co-review with an experienced pathologist. Photographs were captured using an Olympus BX43 microscope equipped with a 10× objective and an Olympus SC50 digital camera. Images were acquired using Olympus cellSens Entry software (version 4.3, Olympus Corporation, Japan). The contrast of the entire IHC image (Fig. 1) was adjusted using the Auto Contrast function in Adobe Photoshop, version 27.4.0 (Adobe Systems, USA), for presentation purposes only. Calcitonin and serotonin stainings were assessed as positive when at least one tumour cell showed intense cytoplasmic staining. Staining results for both hormones were categorised as follows: i) ≥50% of tumour cells positive, ii) 5–50% of tumour cells positive, iii) <5% of tumour cells positive and iv) negative staining. Calcitonin staining was called diffuse when immunoreactivity was observed in more than 50% of tumour cells. Additional stainings for CgA, synaptophysin and TTF-1 were not performed and slides were not re-evaluated, as these markers had already been assessed in nearly all patients as part of the routine clinical diagnostic procedures. Patients in whom hormone measurements were not performed at diagnosis were not included in the analysis for comparisons of positive IHC staining and elevated hormone levels.

### Statistical analysis

Data were summarised using descriptive statistics. Non-normally distributed data and variables with outliers were reported as medians with 25th–75th percentile range or minimum–maximum values. Spearman’s correlation test was used for correlations between variables. Kaplan–Meier plots were used for survival analysis, and the log-rank test was used for comparison. Cox proportional regression was performed for the estimation of hazard ratios (HRs) and confidence intervals (CIs). Associations between categorical variables were examined using the cross-tabulation and Chi-square or Fisher’s exact tests. The defined event was death from any cause. OS was defined as the interval from diagnosis to death; patients without an event were censored at the date of last follow-up (30 April 2025). The level of statistical significance was set to 0.05. All statistical analyses were performed using IBM SPSS Statistics, version 30 (IBM Corp., USA).

## Results

The clinical characteristics of the cohort are summarised in Table 1. Among 167 patients, there were 46 males and 121 females, *P* < 0.001. The median age at tumour diagnosis was 68 (min–max: 15–85) years. A significant difference was observed regarding median age at diagnosis and histological type of the tumour: TC 62 years (min–max: 19–81), AC 68 years (15–85) and LCNEC 71 years (68–78), *P* = 0.001. Sixty-six patients (39.5%) had a history of smoking exceeding 10 pack-years, with predominance among males (51% male vs 37% female). Of all patients in the cohort, 57 had TC (34%), 83 AC (50%), 7 LCNEC (4%), and 20 carcinoid-NOS (12%).

**Table 1:** Baseline characteristics of the cohort.

Variables	Total number of patients *n* = 167				
	TC = 57	AC = 83	NOS = 20	LCNEC = 7	*P*-value*
Female, *n* = 121	70% (40/57)	76% (63/83)	60% (12/20)	86% (6/7)	0.579
Male, *n* = 46	30% (17/57)	24% (20/83)	40% (8/20)	14% (1/7)	
Median age at diagnosis (years)	62 (52–71)	68 (60–74)	72.5 (69–77)	71 (68–78)	*0.001*
Smoking (≥10 pack years)	40% (23/57)	37% (29/83)	45% (9/20)	71% (5/7)	0.348
Surgery for primary	100% (57/57)	49% (41/83)	0%	29% (2/7)	*<0.001*
Primary tumour, median size (mm)	14 (11–21)	22 (15–36)	22 (19–34)	30 (23–42)	*<0.001*
Median Ki-67 at diagnosis (%)	2 (1–3)	10 (7–17)	4 (1–6)	35 (30–80)	*<0.001*
Mitotic count/2 mm^2^					
• <2	100% (57/57)	24% (20/83)	80% (16/20)	0%	*<0.001*
• 2–10	0%	58% (48/83)	0%	29% (2/7)	
• >10	0%	2% (2/83)	0%	29% (2/7)	
• *ND*	0%	16% (13/83)	20% (4/20)	43% (3/7)	
Necrosis	0%	43% (36/83)	0%	71% (5/7)	*<0.001*
Residual tumour at last follow-up	2% (10^†^/57)	87% (72/83)	100% (20/20)	100% (7/7)	*<0.001*
Relapse after surgery of primary	9% (5/57)	58% (24/41)	*N/A*	100% (2/2)	*<0.001*
Median time to relapse (months)	47 (26–115)	31.5 (2–117)	*N/A*	3.5 (1–6)	0.052
Distal metastases at diagnosis	2% (1/57)	55% (46/83)	90% (18/20)	71% (5/7)	*<0.001*

AC, atypical carcinoid; LCNEC, large cell neuroendocrine carcinoma; *N/A*, not applicable; *ND*, not done; NOS, carcinoid not otherwise specified; TC, typical carcinoid.

Medians are accompanied by the 25th–75th percentile ranges for age, size of primary tumour and Ki-67 and by minimum–maximum for time to relapse.

* For *P*-value, only tumours with definitive histological diagnosis (TC, AC and LCNEC) are compared. *P*-values shown in italics are statistically significant (*P* < 0.05)

^†^ Four of 10 patients with TC were classified as having residual disease due to multiple lung nodules >10 mm at diagnosis, histologically confirmed diffuse idiopathic pulmonary neuroendocrine cell hyperplasia and progressive growth during follow-up. One patient who was operated on for the primary tumour had a solitary liver metastasis at diagnosis.

Seventy patients (42%) had distal metastases at the time of diagnosis. One hundred patients (60%) were operated on for their primary tumour, and among them, five had evidence of distal metastases at the time of surgery. Thirty-one patients had a recurrence after a median time of 30 months (min–max: 1–117). Time to recurrence was negatively correlated with Ki-67 in the primary tumour specimen (*ρ* = −0.382, *P* = 0.034) and was significantly shorter in tumours with the presence of necrosis (14 vs 47 months, *P* = 0.017). Twenty-nine of the radically operated patients had lymph node metastases at diagnosis: 12/57 TC (21%), 15/41 AC (37%) and 2/2 LCNEC (100%), *P* = 0.020, and almost half of them (*n* = 14) had a recurrence of the tumour. At the end of follow-up, 109 patients (65%) had remaining tumour disease (36 alive and 73 deceased).

### Hormones

At least one hormone measurement was available in 127 patients. 5-HIAA was measured in 127 patients, calcitonin and ACTH in 86, gastrin in 60, and vasoactive intestinal peptide (VIP) in 26. Seventy-five patients had normal hormone levels, while 52 secreted one (*n* = 34) or multiple (*n* = 18) hormones. A summary of hormone secretion is shown in Table 2. Thirty-eight of the hormone-secreting tumours produced serotonin, and 16 of them were non-functioning without symptoms of a carcinoid syndrome. Patients with elevated 5-HIAA levels were not routinely screened for carcinoid heart disease (CHD) – echocardiography was performed only when clinically indicated. CHD was identified in 1 of 21 examined patients. Twenty patients had elevated calcitonin levels. In 14 of 20 patients with elevated calcitonin, other elevated hormones were also found, compared with 14 of 38 patients with serotonin-secreting tumours. Among patients with isolated elevated calcitonin levels, 2 of 6 reported flushing. High ACTH levels together with clinical features of severe Cushing’s syndrome were observed in 13 patients. One tumour was assessed as gastrin-secreting based on positive gastrin and TTF-1 IHC staining in the primary lung tumour, hormone-induced symptoms and a clear increase in circulating gastrin levels during disease progression. In the remaining nine patients, elevated gastrin levels were considered depending on proton-pump inhibitor therapy and/or atrophic gastritis. VIP levels were elevated in one patient without any hormone-related symptoms.

**Table 2:** Number of hormone-secreting tumours and secretory patterns in different tumour subgroups of neuroendocrine neoplasms of lung, based on circulating hormones.

	TC	AC	NOS	LCNEC
Secreting, total, *n* = 52	7	27	16	2
Serotonin, total, *n* = 38	3	20	14	1
Calcitonin, total, *n* = 20	3	10	6	1
ACTH, total, *n* = 13	2	5	5	1
Serotonin, single, *n* = 24	3	13	7	1
Calcitonin, single, *n* = 6	2	3	1	-
ACTH, single, *n* = 4	1	2	1	-
Serotonin + calcitonin, *n* = 7	-	4	3	-
Serotonin + ACTH, *n* = 2	-	-	2	-
Calcitonin + ACTH, *n* = 2	1	-	-	1
Serotonin + ACTH + calcitonin, *n* = 5	-	3	2	-
Gastrin, *n* = 1	-	1	-	-
VIP, *n* = 1	-	1	-	-

AC, atypical carcinoid; ACTH, adrenocortical hormone; LCNEC, large cell neuroendocrine carcinoma; NOS, carcinoid not otherwise specified; TC, typical carcinoid; VIP, vasoactive intestinal peptide.

Both 5-HIAA and calcitonin levels increased significantly at tumour progression: 5-HIAA increased from 2 × UNL (min–max: 1–101 × UNL) to 7 × UNL (1–101 × UNL) (*P* < 0.001), and median calcitonin levels increased from 14 pmol/L (min–max: 2–777) to 20 pmol/L (3–777) (*P* = 0.003). Among the 27 patients with recurrence after surgery and available hormone assessments, 12 (44%) had increased hormone levels.

### Immunohistochemistry

In 159 patients, tumour tissue was stained for CgA and all were positive. Synaptophysin was positive in 160 patients and negative in one, while TTF-1 was stained in 162 patients and positive in 132.

New stainings for calcitonin and serotonin were performed in 108 patients (TC = 48; AC = 49; LCNEC = 3; NOS = 8); see Tables 3 and 4 for summaries. Calcitonin IHC staining was positive in 45/108 tumours (42%), while 17 (16%) stained positive for serotonin; see Fig. 1. There was no difference in hormone expression between TC and AC. More than 50% of tumour cells stained positive in 16 of 45 calcitonin-positive specimens, while this was observed in only 2/17 serotonin-positive specimens.

**Table 3:** Immunohistochemical characteristics of neuroendocrine neoplasms of the lung depending on tumour morphology.

Markers	TC pos/total *n*	AC pos/total *n*	NOS pos/total *n*	LCNEC pos/total *n*
CgA (*n* = 159)	56/56	78/78	19/19	6/6
Synaptophysin (*n* = 161)	57/57	77/77	20/20	6/7
TTF-1 (*n* = 162)	46/56	65/80	15/20	6/6
Ki-67 (*n* = 166)				
• <5%	51	11	11	0
• 5–20%	6	61	8	0
• 21–55%	0	11	0	5
• >55%	0	0	0	2
Serotonin (*n* = 108)	10/48	7/49	0/8	0/3
Calcitonin (*n* = 108)	23/48	18/49	2/6	2/3

AC, atypical carcinoid; LCNEC, large cell neuroendocrine carcinoma; NOS, carcinoid not otherwise specified; TC, typical carcinoid.

**Table 4:** Results from the immunohistochemical scorings for serotonin and calcitonin in neuroendocrine neoplasms of the lung.

	Serotonin, *n* = 108 (%)		Calcitonin, *n* = 108 (%)	
	Pos.	Neg.	Pos.	Neg.
Total *n* (%)	17 (16)	91 (84)	45 (42)	63 (58)
• Pos ≥ 50%	2 (12)	*N/A*	16 (35)	*N/A*
• Pos 5–50%	5 (29)		17 (38)	
• Pos < 5%	10 (59)		12 (27)	

*N/A*, not applicable.

Among the 108 patients with IHC staining for calcitonin, 34 had corresponding calcitonin measurements in blood at diagnosis, whereas 43/108 with serotonin IHC staining had corresponding 5-HIAA measurements. Elevated 5-HIAA levels at diagnosis were identified in 3/5 patients with serotonin-positive tumour tissue and in 13 of 38 patients with serotonin-negative tumours (34%). No difference was observed between the groups (OR: 2.88, *P* = 0.34). In contrast, calcitonin expression was significantly associated with elevated hormone levels: 9/16 calcitonin-positive tumours (56%) had elevated circulating hormones, compared to 1/18 calcitonin-negative tumours (6%) (OR: 21.86, *P* = 0.002). At diagnosis, tumours with negative IHC staining for both serotonin and calcitonin had significantly higher Ki-67 compared to hormone-positive tumours (median Ki-67: 6 vs 2%; *P* = 0.009). In contrast, when Ki-67 was analysed in relation to circulating hormones, the highest median Ki-67 was found in tumours secreting both serotonin and calcitonin (10%), followed by serotonin-secreting tumours (7%), calcitonin-secreting tumours (2%) and non-secreting tumours (3%) (*P* = 0.134). IHC positivity for calcitonin and/or serotonin was not associated with a higher risk of tumour recurrence after surgery.

### Survival

At the end of the study period, 93 patients were still alive (54/57 = TC; 31/83 = AC; 0/7 = LCNEC; 8/20 = NOS). The median OS in the whole cohort was 101 months (95% CI: 86–116) with a median 5-year survival rate of 66%. Tumour morphology had a negative prognostic impact, *P* < 0.001: the median OS was 72 months (95% CI: 50–94) for AC, 18 months (95% CI: 3–33) for LCNEC and 69 months (95% CI: 37–101) for NOS. For TC, the median OS could not be estimated because too few events occurred during follow-up.

Higher age at diagnosis, smoking, poorer differentiation and presence of local lymph node metastases were negative prognostic factors in univariate Cox regression analysis. In metastatic disease, patients with hormone-secreting tumours had a significantly shorter OS compared to those without hormone secretion, 62 vs 124 months, *P* = 0.024. Furthermore, concomitant multiple hormone secretion was associated with a shorter OS compared to secretion of only one hormone or to non-secreting tumours, *P* = 0.002, 33 vs 69 vs 124 months. Multiple hormone secretion remained a statistically significant negative prognostic factor in multivariate Cox regression analysis, *P* = 0.042 (HR: 2.8 (95% CI: 1.0–7.7)); see Table 5.

**Table 5:** Univariate and multivariate analysis of prognostic factors for survival in neuroendocrine neoplasms of the lung.

Variable	Median OS (months*)	HR (95% CI)	*P*-value^‡^
**Univariate analysis for the whole cohort**			
Median age (>68 years vs ≤ 68 years)	49 vs *N/D*^†^	3.3 (2.0–5.3)	*0.001*
Gender (female vs male)	109 vs 69	0.7 (0.4–1.2)	0.175
Smoking (no vs yes)	124 vs 72	0.5 (0.3–0.9)	*0.011*
Morphology (LCNEC vs AC vs TC)	18 vs 72 vs *N/D*^†^	7.6 (4.5–13.0)	*<0.001*
Lymph node metastases at surgery (no vs yes)	150 vs 121*	0.4 (0.2–1.0)	*0.046*
**Univariate analysis for patients with remaining tumour disease at last follow-up**			
Hormone-secreting tumour (no vs yes)	124 vs 62	0.4 (0.2–0.9)	*0.024*
Multiple hormones vs one vs none	33 vs 69 vs 124	2.1 (1.3–3.5)	*0.002*
**Multivariate analysis for patients with remaining tumour disease at last follow-up**			
Median age (>68 years vs < 68 years)	*N/A*	2.4 (1.0–6.0)	0.050
Smoking (no vs yes)		0.6 (0.3–1.5)	0.339
Morphology (LCNEC vs AC vs TC)		3.2 (1.0–9.4)	*0.033*
Hormone-secreting tumour (yes vs no)		1.5 (0.3–7.9)	0.623
Multiple hormones vs one vs none		2.8 (1.0–7.7)	*0.042*

AC, atypical carcinoid; LCNEC, large cell neuroendocrine carcinoma; *N/A*, not applicable; OS, overall survival; TC, typical carcinoid.

Hazard ratio (HR) and 95% confidence intervals (CIs) obtained from Cox regression models.

* If median OS is not defined at all because per cent survival in all groups at the end of the follow-up exceeds 50%, the mean survival is shown instead.

^†^ Median survival time and/or CI is not defined because per cent survival at the end of the follow-up exceeds 50%.

^‡^ *P*-values shown in italics are statistically significant (*P* < 0.05).

**Figure 2:**
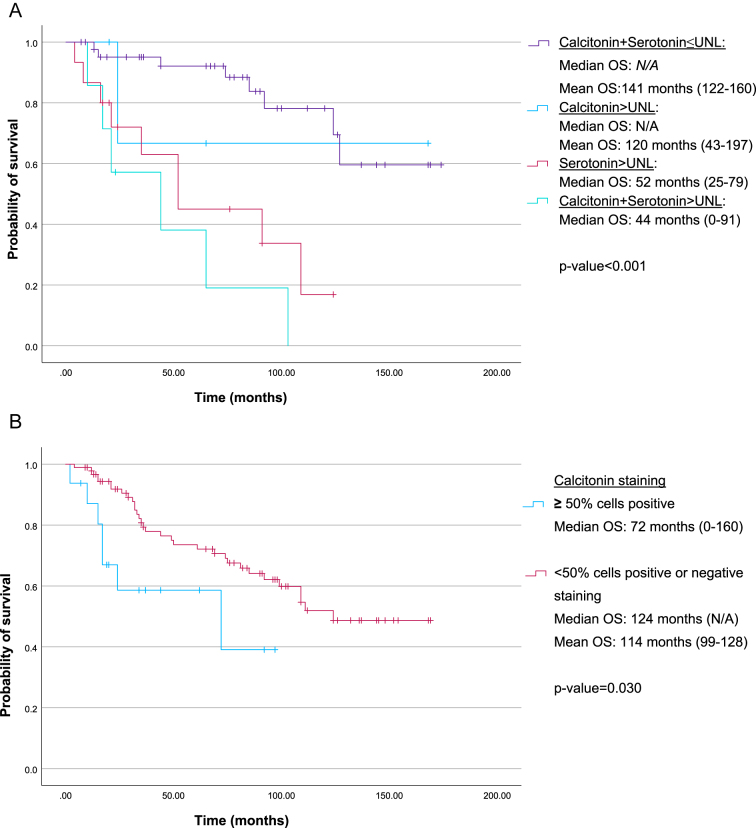
Kaplan–Meier overall survival (OS) curves for patients with neuroendocrine neoplasms of the lung stratified according to circulating calcitonin and serotonin levels, as well as tumour calcitonin expression determined by immunohistochemistry. (A) Patients with non-secreting tumours compared with those with single-hormone secretion (either calcitonin or serotonin) or co-secretion of both. (B) Positivity for calcitonin in immunohistochemical staining in >50% of tumour cells, compared to less or absence of positivity. OS is represented as median survival in months with 95% confidence intervals (CIs). If median OS or confidence intervals are not defined because per cent survival at the end of the follow-up exceeds 50% (N/A), the mean survival is shown instead. A full colour version of this figure is available at https://doi.org/10.1530/ERC-26-0142.

When estimating OS in patients with available measurements for both calcitonin and serotonin, the shortest OS was observed in tumours secreting both calcitonin and serotonin with a median OS of 44 months (95% CI: 0.0–91.1) and the longest OS was observed in patients without secretion of either hormone, mean OS: 141 months (95% CI: 121.7–159.9, *P* < 0.001); see Fig. 2A.

Diffuse calcitonin expression in more than 50% of tumour cells compared to lower or absent expression was associated with poorer survival, *P* = 0.036 (HR: 0.4 (95% CI: 0.2–0.9)); see Fig. 2B. Similar analysis for serotonin was not possible since only two cases of 108 expressed serotonin in >50% of tumour cells.

## Discussion

To our knowledge, this is the first study demonstrating hormone secretion in lung-NENs as a negative prognostic factor. Serotonin secretion, whether occurring alone or in combination with calcitonin secretion, was strongly associated with poorer outcome. Furthermore, we identified high calcitonin expression in tumour tissue as a negative prognostic factor.

Thirty-one per cent of all patients in our cohort had elevated hormone levels, which are higher than reported previously (16). When excluding patients without any hormone measurements, the percentage of patients with elevated hormone levels increased to 57%. This high prevalence could be explained by centre-specific bias, with a systematic hormone testing performed in our cohort. The true prevalence of hormone-secretion in lung-NENs may be underestimated due to the lack of previous recommendations for routine testing in the absence of hormonal syndrome. Elevated 5-HIAA levels were observed in 38 of 127 tested patients, exceeding the number reported by ENETS review of literature (10). Interestingly, in a previously reported cohort of 162 patients with metastatic lung-NENs only, carcinoid syndrome was found in 38% of patients (28). This finding supports our results. The second most frequently elevated hormone in our cohort was calcitonin, which was found in 20 of 86 tested patients, usually in combination with the secretion of other hormones. Reports of hypercalcitoninaemia in lung-NENs are very rare and largely limited to single case reports or to studies describing calcitonin secretion in cohorts of NENs of both pulmonary and gastrointestinal origin (15, 29).

Several studies have reported IHC expression of hormones in lung-NENs (22, 30). However, to our knowledge, no previous investigations have investigated the relationship between tissue hormone expression and corresponding circulating hormone levels. We found calcitonin positivity (42%) to be more common than serotonin positivity (16%), which is in contrast to the findings of Vesterinen *et al*., where serotonin expression was more frequent (22). However, their cohort of 133 patients contained 75% of typical carcinoids, whereas our cohort demonstrated an even distribution between TC and AC. Similar to our observations, they showed that serotonin positivity in a majority of cells was only detected in few samples, while a more common staining pattern was single cell positivity (22).

Among serotonin-negative tumours, 34% had elevated circulating 5-HIAA levels, while only 6% of calcitonin-negative tumours were hormone-secreting. These findings may indicate that calcitonin expression in tumour tissue is a reliable marker of hormone secretion, whereas serotonin expression in the tumour appears insufficient to predict elevated circulating hormone levels. The lack of serotonin expression in tumours despite elevated 5-HIAA levels may have several explanations, including low intracellular serotonin storage due to rapid secretion; reduced L-amino acid decarboxylase activity leading to secretion of 5-HTP rather than serotonin (31, 32); or expression of monoamine oxidase A within tumour cells resulting in an intracellular metabolism of serotonin to 5-HIAA. However, no studies have investigated these hypothesised mechanisms in lung-NENs. The discrepancy between immunohistochemical findings and hormonal levels in blood may also be explained by tumour heterogeneity in NENs and variability in tissue sampling sites.

Vesterinen *et al.* did not find any association between IHC expression of serotonin or calcitonin in lung-NENs and patient’s outcome (22). In contrast, we found that calcitonin expression in more than 50% of tumour cells, compared with lower or absent expression, was associated with poorer survival. There are few reports supporting serum calcitonin as a negative prognostic factor in NEN, but none specifically investigating lung-NENs (15, 29).

Our findings indicate that hormone secretion in lung-NENs may be an important predictor for poorer outcome. The presence of single or multiple hormone secretion had a significant negative impact on median OS compared with non-secreting tumours. Tumours secreting serotonin, either alone or in combination with calcitonin, were associated with poorer outcome than those with isolated calcitonin secretion or no hormonal secretion. Furthermore, tumours secreting both hormones tend to have higher Ki-67. Forty-one per cent of patients with elevated 5-HIAA did not report characteristic symptoms for carcinoid syndrome. Similar discrepancies between 5-HIAA levels and symptoms have also been reported in cohorts of pancreatic NENs (31, 32). The recently published ENETS guidance paper has, for the first time, recommended routine assessment of 5-HIAA in all patients with metastatic lung NENs, as elevated levels have been reported in asymptomatic patients and are associated with an increased risk of carcinoid heart disease (33).

The evidence regarding the prognostic impact of serotonin secretion on OS in NENs is limited and contradictory. Several studies have reported a significantly shorter OS in patients with serotonin-secreting NENs (34, 35, 36), whereas others have suggested that serotonin has limited prognostic value once additional biomarkers and tumour grade are taken into account (37). These studies have been conducted predominantly in gastrointestinal NENs. Some reports have identified ACTH secretion – either alone or in combination with other hormones – as a negative prognostic factor in lung-NENs (17, 18). In addition, metachronous hormone-induced syndromes have been described in the context of progressive pancreatic NENs (19, 20), and in our study, multiple hormone secretion emerged as an independent negative prognostic factor. Tumour morphology was the strongest prognostic factor in our cohort, with LCNEC having the poorest outcome.

In conclusion, this study provides the first evidence that hormone secretion in lung-NENs, particularly serotonin, may be an independent risk factor for reduced OS. Moreover, strong and diffuse calcitonin expression in tumour tissue is associated with poorer clinical outcome and correlates with circulating calcitonin levels. Based on these findings, we suggest that, in addition to routine assessment of 5-HIAA, calcitonin measurement may also be considered at diagnosis and during disease progression and that calcitonin immunohistochemical staining should be included in the evaluation of metastatic lung-NENs. Patients with hormone-secreting lung-NENs should be monitored closely and may benefit from more intensive therapeutic strategies. Multicentre studies with large, prospective cohorts are needed to validate our observations regarding those previously understudied prognostic factors in lung-NENs.

## Declaration of interest

The authors declare that there are no conflicts of interest that could be perceived as prejudicing the impartiality of the research reported.

## Funding

This work was supported by the Swedish Cancer Society (grant number 23 2890). The funders had no role in study design, data collection and analysis, decision to publish or preparation of the manuscript.

## Author contribution statement

ETJ, SW and IL conceptualised the project and designed the study. IL and EL acquired all the data. IL analysed the data and wrote the main manuscript. MG contributed to the statistical analysis. IL and AH evaluated IHC stainings. All the authors interpreted the data, drafted the article, revised it and approved the final version. ETJ, SW and MG supervised the study.

## Ethical approval

All procedures performed in this study were in accordance with the ethical standards of the institutional and/or national research committee and with the 1964 Declaration of Helsinki and its later amendments or comparable ethical standards. This study was approved by the local ethics committee, Swedish Ethical Review Authority (EPM) in Uppsala, Sweden (EPM nr. 2024-08216-01).

## Informed consent

Written informed consent was obtained from all living participants in accordance with the Declaration of Helsinki. The requirement for informed consent for deceased participants was waived by the local ethics committee.
